# What Are the Risk Factors Associated with Urinary Retention after Orthopaedic Surgery?

**DOI:** 10.1155/2015/613216

**Published:** 2015-02-18

**Authors:** Ki Hyuk Sung, Kyoung Min Lee, Chin Youb Chung, Soon-Sun Kwon, Seung Yeol Lee, Yoon Seong Ban, Moon Seok Park

**Affiliations:** ^1^Department of Orthopaedic Surgery, Myongji Hospital, 55 Hwasu-ro, 14 Beon-gil, Deokyang-gu, Goyang, Gyeonggi-do 412-826, Republic of Korea; ^2^Department of Orthopaedic Surgery, Seoul National University Bundang Hospital, 300 Gumi-dong, Bundang-gu, Seongnam, Gyeonggi-do 463-707, Republic of Korea; ^3^Biomedical Research Institute, Seoul National University Bundang Hospital, 300 Gumi-dong, Bundang-gu, Seongnam, Gyeonggi-do 463-707, Republic of Korea; ^4^Department of Orthopaedic Surgery, Ewha Womans University Mokdong Hospital, 1071 Anyangcheon-ro, Yangcheon-gu, Seoul 158-710, Republic of Korea

## Abstract

This study investigates the overall rate of urinary retention in a large cohort of unselected orthopaedic patients who had either general or regional anesthesia and defines the risk factors for postoperative urinary retention in that cohort of patients. A total of 15,681 patients who underwent major orthopaedic surgery with general or spinal/epidural anesthesia were included. Postoperative urinary retention was defined as any patient who required a postoperative consultation to the urologic department regarding voiding difficulty. Age at surgery, sex, type of surgery, medical history including hypertension and diabetes mellitus, and type of anesthesia were analyzed as potential predictor variables. There were 365 postoperative patients who required urology consults for urinary retention (2.3%). Older age at surgery (OR, 1.035; *P* < 0.0001), male sex (OR, 1.522; *P* = 0.0004), type of surgery (OR, 1.506; *P* = 0.0009), history of hypertension (OR, 1.288; *P* = 0.0436), and history of diabetes mellitus (OR, 2.038; *P* < 0.0001) were risk factors for urinary retention after orthopaedic surgery. Advanced age, male sex, joint replacement surgery, history of hypertension, and diabetes mellitus significantly increased the risk of urinary retention. In patients with these risk factors, careful postoperative urological management should be performed.

## 1. Introduction

Urinary retention is a common complication after surgery and can be a significant source of patient anxiety and discomfort. Urinary retention results in a longer hospital stay, increased hospital costs, and sometimes additional morbidity [1]; bladder overdistension may lead to the reduction of the contractile function of detrusor muscle and chronic impairment of bladder emptying or atony [2]. By contrast, urethral catheterization should only be performed where needed, as it can cause urinary tract infection, urethral stricture, and the need for additional surgery [3]. Identifying which patients need catheterization and which ones do not is therefore important.

Reported rates of postoperative urinary retention vary widely. Orthopedic patients have an increased risk of postoperative urinary retention (8% to 55%) [4–7] compared with that of other surgical patients. However, these findings were confined to total joint arthroplasty of the knee and hip. Type of anesthesia [8, 9], postoperative pain [10], use of analgesics and opiates [11], volume of intravenous fluid during the perioperative period [12], age [13], sex [4, 5], and concomitant medical disease [6] also have been associated with the development of postoperative urinary retention. However, to date, no study investigated the frequency of urinary retention after orthopaedic surgery in a large, unselected patient cohort. In addition, there is uncertainty regarding which factors may predispose patients to urinary retention after orthopaedic surgery.

Therefore, this study was performed in a large cohort of unselected patients undergoing regional or general anesthesia before orthopaedic surgery but who did not have a urinary catheter placed in advance of the surgical procedure to address the following questions: (1) what is the overall rate of urinary retention after orthopaedic surgery and (2) what risk factors, if any, predispose an orthopaedic patient to postoperative urinary retention?

## 2. Materials and Methods

This retrospective study was approved by the institutional review board at our hospital, a tertiary referral center. The inclusion criteria were (1) inpatients who underwent orthopaedic surgery between 2003 and 2013, (2) who underwent surgery under general or spinal/epidural anesthesia, and (3) who had information about postoperative urination in nursing electrical medical records (EMRs). Patients who had a Foley catheter placed prior to or during the surgery were excluded.

From the EMR reviews, patients' age at surgery, sex, type of surgery, medical history including hypertension (HTN) and diabetes mellitus (DM), type of anesthesia, and development of postoperative urinary retention were obtained. Our institution, a tertiary referral center, achieved Healthcare Information and Management Systems Society Analytics Stage 7 for an EMR system [14]. Nursing records were based on the International Classification for Nursing Practice (ICNP), which was a nursing classification for nursing diagnoses, interventions, and outcomes being developed by the International Council of Nurses (ICN) [15].

If the patients were unable to spontaneously void when the bladder became distended, intermittent catheterization or Foley catheterization was performed by an orthopaedic resident, intern, or nurse. For these patients, urologic consultation was performed routinely. Postoperative urinary retention was defined in this study as the need for postoperative consultation to the urology department regarding voiding difficulty. Patients with urinary retention were treated with intermittent catheterization or Foley catheterization; regardless of the approach used, all of those patients were classified as having had urinary retention for purposes of analysis in this study. Type of surgery was classified into joint arthroplasty and other types of surgery. Joint arthroplasty included hemiarthroplasty, total joint arthroplasty, and revision procedures. “Other types of surgery” included all other orthopaedic patients who had either a general or neuraxial anesthetic during the period of study.

Patients were divided into the retention group and the nonretention group according to development of postoperative urinary retention as defined earlier. Patient demographics in the retention group were compared with those in the nonretention group. The risk factors for the development of urinary retention after orthopaedic surgery were analyzed.

*Statistical Analysis.* For the purpose of statistical independence, only data from the first procedure of patients who underwent orthopaedic surgery several times were included for statistical analysis [16]. Descriptive statistics were used to summarize patient demographics. An independent *t*-test or chi square test was used to compare the preoperative demographics between the retention group and nonretention group. Multivariate logistic regression analysis was used to analyze the significant contributing factors for the development of urinary retention after orthopaedic surgery. Statistical analyses were conducted using SPSS for Windows (Version 18.0; SPSS, Chicago, IL, USA), and null hypotheses of no difference were rejected if *P* values were <0.05.

## 3. Results

**Figure 1:**
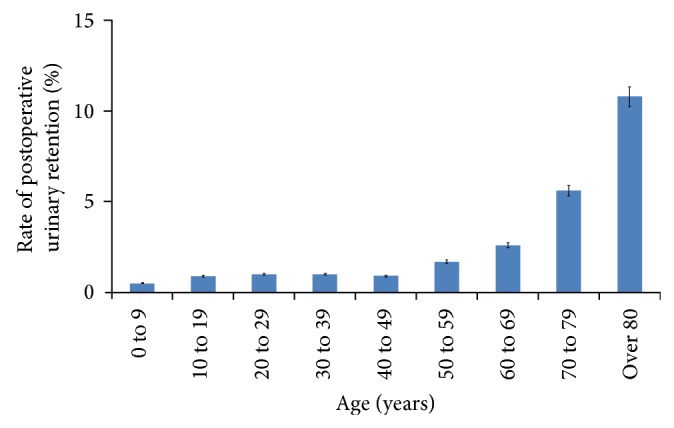
This plot depicts the rate of postoperative urinary retention according to age.

Since 2003, 19,079 inpatients underwent orthopaedic surgery under general or spinal/epidural anesthesia. Three thousand three hundred ninety-eight patients who underwent Foley catheterization prior to or during the surgery were excluded according to our predefined criteria. Thus, 15,681 patients were enrolled in this study, including 7798 males and 7883 females. Mean age at surgery was 45 ± 23 years (range, 0–107 years). A total of 2.3% of patients (365 out of 15,681) experienced urinary retention (Table 1). The rate of postoperative urinary retention was highest at those older than 80 years (11.0%) followed by those in their 70s (5.7%) and 60s (2.7%) (Figure 1).

Patient demographics including age, gender, type of surgery, history of HTN, and history of DM in the retention group differed significantly from the nonretention group. However, there was no difference in type of anesthesia between two groups (Table 2).

Older age (odds ratio (OR), 1.035; 95% confidence interval (CI), 1.028–1.043; *P* < 0.0001), male sex (OR, 1.522; 95% CI, 1.207–1.919; *P* = 0.0004), joint replacement surgery (OR, 1.506; 95% CI, 1.183–1.917; *P* = 0.0009), history of hypertension (OR, 1.288; 95% CI, 1.007–1.648; *P* = 0.0436), and history of diabetes mellitus (OR, 2.038; 95% CI, 1.591–2.611; *P* < 0.0001) were associated with urinary retention on our multivariate analysis (Table 3).

## 4. Discussion

The aim of the current study was to investigate the overall rate of urinary retention after orthopaedic surgery and to identify the risk factors for postoperative urinary retention. This study showed that the overall rate of postoperative urinary retention was 2.3% after orthopaedic surgery and that older age at surgery, male sex, joint replacement surgery, and histories of hypertension and diabetes mellitus were associated with an increasing risk of postoperative urinary retention.

There were several limitations to this study. First, this was a retrospective study in its design, and the development of urinary retention was by identifying urological consultation from medical record review and not by objective method such as bladder scan; if anything, therefore, the risk of urinary retention might have been underestimated by our analysis, but there is no reason to believe this issue would have affected the risk factors differently from one another. Second, patients with a Foley catheter placed prior to or during surgery were excluded from this study, which might have resulted in data bias. However, this study focused on the patient group that did not need preoperative catheterization and investigated the incidence and risk factors of postoperative urinary retention in this group. Third, other factors including a history of BPH or urinary retention, perioperative pharmacologic agents, and duration of surgical procedures, which might be confounders, were not included when analyzing the risk factors in this study. Further study including these factors is required.

We found that 2.3% of orthopaedic patients experienced postoperative urinary retention, and the frequency in our study was lower than the previously reported frequency of 8–55% [4–7, 11]. Our cohort included patients aged 0–107 years who underwent all types of orthopaedic procedure. However, previous studies included elderly patients who underwent joint replacement surgery, which might cause higher incidence of urinary retention than current study. Therefore, we believe that our study represents overall incidence of urinary retention after orthopaedic surgery.

Previous studies reported that type of anesthesia, volume of intravenous fluids, age, sex, pharmacological therapy, use of opioids, and history of HTN were significant factors affecting the development of postoperative urinary retention. Our study supported some but not all of those findings.

Several studies have shown the association between spinal/epidural anesthesia and postoperative urinary retention [9, 17, 18]. On the other hand, some authors concluded that the type of anesthetic technique did not influence the incidence of retention [6, 19], which was consistent with the result of our study. With the use of intrathecal local anesthetics, the duration of detrusor blockade allows the bladder volume to exceed preoperative bladder capacity, and urinary retention develops [20]. General anesthetics cause bladder atony by acting as smooth muscle relaxants and by interfering with autonomic regulation of detrusor tone, which also can develop urinary retention [8].

A number of studies have demonstrated that patients' age was the significant risk factor for postoperative urinary retention [9, 12, 13, 17, 21]. Our study also showed that the risk of postoperative urinary retention was 1.4 times higher as age increased by 10 years. It has been reported that detrusor function deteriorates progressively with advancing age and that bladder sensation declines with advancing age [22]. The aged may also be more susceptible to the negative urodynamic effects of analgesic and anesthetic agents because many of these drugs have a prolonged duration of action in the elderly. In this study, men had a 1.5-fold increased risk of urinary retention after orthopaedic surgery.

Several studies have confirmed that older males are at an increased risk for the development of urinary retention, which concurs with our results [4, 5, 18, 21]. Mechanical blockage of urinary outflow secondary to benign prostatic hyperplasia or urethral stricture may be the primary source of bladder morbidity in elderly men.

Our results showed that the risk of postoperative urinary retention was 1.3 times higher in patients with HTN and two times higher in patients with DM. Izard et al. [6] demonstrated that a history of HTN predicted increased risk of urinary retention after major joint replacement surgery. In a previous study, the use of beta-blockers was associated with an increased risk of postoperative urinary retention [23]. Therefore, common use of beta-blockers in patients with HTN may explain our findings. A previous study found that patients with diabetes are prone to the development of postoperative urinary retention, consistent with the current study [24]. DM, one of the most common comorbid diseases in older patients, is often associated with impairment in bladder sensation, increased bladder capacity, and decreased contractility [25].

Our study demonstrated that patients who underwent joint arthroplasty had a 1.5-fold increased risk of developing postoperative urinary retention compared to patients who underwent other types of surgery. We believe that various factors including increased age, prolonged surgical time, concomitant medical illness, administration of greater volumes of intravenous fluids, and higher doses of opioids and anesthetic agent in patients who underwent joint arthroplasty might lead to this result. Further study on this result is needed.

Increased age, male sex, joint replacement surgery, and history of hypertension or diabetes were associated with an increased risk of urinary retention after orthopaedic surgery. Therefore, in patients with these risk factors, careful postoperative urological management should be performed. Further research is required to determine the optimal duration of catheterization to minimize the risk of urinary tract infection.

## Figures and Tables

**Table 1:** Patient demographics and characteristics.

Demographic or characteristic	Number of patients
Sex (male/ female)	7798/7883
Age at surgery (years)	45.2 ± 23.1 (range, 0–107)
Type of anesthesia (general/spinal or epidural)	7372/8309
Type of surgery (joint arthroplasty/other types of surgery)	3784/11,897
Medical history of HTN	3630
Medical history of DM	1610
Consultation to urology department	752
Postoperative urinary retention	365
Voiding problem^*^	234
Urinary tract infection	36
Genitourinary tract disease^†^	39
Genital organs problem	39
Preoperative voiding difficulty	20
Trauma	19

^*^Voiding problems include frequency, hematuria, dysuria, nocturia, incontinence, and urgency; ^†^genitourinary tract diseases include benign prostate hypertrophy, malignancy, and urinary stone; HTN = hypertension; DM = diabetes mellitus.

**Table 2:** Comparison of patient demographics between the urinary retention group and nonretention group.

Variables	Retention group (365 patients)	Nonretention group (15831 patients)	*P* value
Age (years)	62.8 ± 20.0	44.7 ± 23.0	<0.001
Sex (male/female)	154/211	7,644/7,672	0.004
Type of anesthesia (general/spinal or epidural)	172/193	7,200/8,116	0.966
Type of surgery (joint arthroplasty versus other types of surgery)	180/185	3,604/11,712	<0.001
History of hypertension (yes/no)	183/182	3,447/11,869	<0.001
History of diabetes mellitus (yes/no)	112/253	1,498/13,818	<0.001

The independent *t*-test or chi square test was used to evaluate the statistical significance in patient demographics between the retention group and nonretention group.

**Table 3:** Risk factors for the urinary retention after orthopaedic surgery.

Variables	Odds ratio	95% CI	*P* value
Age at surgery	1.035	1.028–1.043	<0.001
Sex (male versus female)	1.522	1.207–1.919	<0.001
Type of surgery (joint arthroplasty versus other types of surgery)	1.506	1.183–1.917	0.001
History of hypertension (yes versus no)	1.288	1.007–1.648	0.044
History of diabetes mellitus (yes versus no)	2.038	1.591–2.611	<0.001

The multivariate logistic regression analysis was used to analyze the significant risk factors for the postoperative urinary retention; CI = confidence interval.
